# Characteristics and predictors of oral cancer knowledge in a predominantly African American community

**DOI:** 10.1371/journal.pone.0177787

**Published:** 2017-05-17

**Authors:** Nosayaba Osazuwa-Peters, Eric Adjei Boakye, Adnan S. Hussaini, Nanthiya Sujijantarat, Rajan N. Ganesh, Matthew Snider, Devin Thompson, Mark A. Varvares

**Affiliations:** 1 Saint Louis University Cancer Center, Saint Louis, MO, United States of America; 2 Saint Louis University, School of Medicine, Department of Otolaryngology-Head and Neck Surgery, Saint Louis, MO, United States of America; 3 Saint Louis University Center for Outcomes Research (SLU*COR*), Saint Louis University, Saint Louis, MO, United States of America; 4 Saint Louis University, School of Medicine, Saint Louis, MO, United States of America; Leibniz Institute for Prvention Research and Epidemiology BIPS, GERMANY

## Abstract

**Purpose:**

To characterize smoking and alcohol use, and to describe predictors of oral cancer knowledge among a predominantly African-American population.

**Methods:**

A cross-sectional study was conducted between September, 2013 among drag racers and fans in East St. Louis. Oral cancer knowledge was derived from combining questionnaire items to form knowledge score. Covariates examined included age, sex, race, marital status, education status, income level, insurance status, tobacco and alcohol use. Adjusted linear regression analysis measured predictors of oral cancer knowledge.

**Results:**

Three hundred and four participants completed questionnaire; 72.7% were African Americans. Smoking rate was 26.7%, alcohol use was 58.3%, and mean knowledge score was 4.60 ± 2.52 out of 17. In final adjusted regression model, oral cancer knowledge was associated with race and education status. Compared with Caucasians, African Americans were 29% less likely to have high oral cancer knowledge (β = -0.71; 95% CI: -1.35, -0.07); and participants with a high school diploma or less were 124% less likely to have high oral cancer knowledge compared with college graduates (β = -1.24; 95% CI: -2.44, -0.41).

**Conclusions:**

There was lower oral cancer knowledge among African Americans and those with low education. The prevalence of smoking was also very high. Understanding predictors of oral cancer knowledge is important in future design of educational interventions specifically targeted towards high-risk group for oral cancer.

## Introduction

There will be an estimated 48,330 new cases of oral cavity & pharynx cancer (or oral cancer) in the United States in 2016, resulting in 9,570 deaths [1]. The causal factors associated with oral cancer are tobacco and excessive alcohol use, as well as human papillomavirus (HPV) infection; HPV being mostly associated with oropharyngeal subset [1–3]. There has been a steady decrease in the incidence of tobacco and alcohol associated oral cancer in the United States, while the HPV-associated oropharynx cancer has increased about 225% in the same time period [4–6]. However, tobacco and alcohol are still associated with at least 75% of all cases of head and neck cancers [7,8], and individuals who smoke and drink heavily have 30 to 48 increased odds of developing oral cancer compared to non-smokers or non-drinkers [1,9].

Although adult smoking rates in the United States is on a decline, coinciding with a decrease in oral cancer incidence, this decrease is not uniform across all social and demographic groups [10,11]. In cities and urban areas across the United States, there remains an increasing incidence of tobacco and alcohol use among African Americans [12]. Smoking rates, significantly higher than the national average have also been reported among fans at National Association for Stock Car Auto Racing (NASCAR) events [13,14].

It is a generally held belief that at least three-quarters of all oral cancers could be prevented as they are mostly associated with modifiable health behaviors, including smoking and alcohol use [8]. Along with primary prevention through avoiding risky behaviors, early detection often leads to better prognosis, and is a key objective in the *Healthy People 2020* initiative [15]. However, early detection is not often realistic for most individuals, only one-third of patients present with early stage oral cancer disease [16]. The United States Preventive Services Task Force (USPSTF) guidelines on cancer screenings state that there is currently no evidence of any mortality benefits associated with mass screening of asymptomatic individuals for oral cancer [17], and recommends that asymptomatic oral cancer screening should be done as part of routine dental or medical check-up. However, there are more than 100 million Americans without dental insurance[18], and access to healthcare is a major driver of disparities in the community [19,20]. This indicates that there remains the need to provide some form of oral cancer screening services to specific populations deemed high-risk who may not have access to healthcare. In Kerala, India, a clinical trial involving almost 100,000 participants demonstrated that there could be some mortality benefits in mass oral cancer screening, especially among high-risk individuals [21]. There has also been other successful mass oral cancer screening programs in New York, and Maryland [22,23], as well as interventions in Italy and England employing the use of educational leaflets in increasing knowledge of oral cancer [24,25]. Compared to the cost of managing oral cancer, community oral cancer screenings and educational measures may be far more cost-effective [24,26]. During community oral cancer screening events, there is also an additional value of providing education and general awareness about oral cancer risk factors [27], since population knowledge levels are suboptimal [28–32], and knowledge level has been shown to be associated with oral cancer screening practices [33–37]. Other than community mass oral cancer screenings, there have also been other studies from Florida, Maryland, North Carolina, and New York that employed telephone-based surveys to elicit awareness of oral cancer, as well as knowledge of oral cancer risk factors (tobacco and alcohol) among whites, blacks, and other racial/ethnic groups [28,33,38,39].

Since urban African American communities, and race car fans both have high smoking rates, we sought to measure smoking rates in the two groups discussed above, as there is presently no study exclusively focused on drag race attendees who are predominantly African Americans. Previous studies on oral cancer and smoking rates among NASCAR fans have been predominated by Caucasians, since the sport has a much larger Caucasian following than African Americans [13,14,40]. We hypothesized that smoking rates will be higher in this community than the national average; and will be associated with educational levels, whereby those highly education will less likely be smokers. We also hypothesized that oral cancer knowledge will be low in this population, especially among African Americans.

The objectives of this study were to characterize smoking and alcohol use, and describe predictors of oral cancer knowledge among a predominantly African-American population of drag racing drivers and their fans.

## Methods

### Study design and participants

This was a cross-sectional study conducted between September 13–14, 2013 among drag racers and fans in East St. Louis. The study used a convenience sample that included drag racers and spectators at the Black Sunday race event hosted by the United Black Drag Racers Association (UBDRA) at Gateway Motorsports Park in East St. Louis. About 3,800 individuals attended the event and oral cancer screening and survey administration was done on September 13 and 14. The UBDRA provided a designated area with tents for different health screenings, including oral head and neck cancer screening. Participants of all races aged 18 years or older were eligible to take part in the study, and eligible individuals were asked to complete a survey as well as receive a complimentary oral head and neck cancer screening. Out of the approximately 3000 possible participants, 303 participants agreed to fill out the questionnaire resulting in a participation rate of about 10% which is similar to previous studies focusing on NASCAR fans [31]. In appreciation for their time, each participant was rewarded with a raffle ticket upon survey completion. Prizes available for raffle were of minimal monetary value, donated to the cancer outreach team by community partners. The Saint Louis University Institutional Review Board approval was obtained prior to beginning data collection and the study was performed in accordance with the Declaration of Helsinki (Protocol ID: 23696, approved on August 30, 2013).

### Data collection

The study employed a previously validated 50-item, paper-based questionnaire adapted from previous studies [22,41–43] which had focused on similar or same population. Sociodemographic factors in the study include age, sex, race (Caucasian, African American, Others—which combines low-frequency responses), marital status (married/cohabitating, not married), education (≤ high school diploma, some college/associate degree, and college graduate or higher), household income (< $20,000; $25,000 –$39,999; $40,000 –$74,999; ≥ $75,000), and insurance status (yes, no). Cigarette smoking and alcohol use were assessed with the questions *“Do you smoke?” and “Do you drink alcohol?”* respectively. Results were coded as yes and no for both variables.

### Knowledge of oral cancer

Participants were asked 17 questions to gauge their knowledge of oral cancer. General questions included such questions as: *“In your opinion*, *oral cancers are more common in which age group?” “In your opinion*, *in which gender are oral cancers more common?”* and *“Does early diagnosis improve recovery from oral cancers?”* Furthermore, basic questions concerning pathophysiology were asked such as *“In your opinion*, *where are the most likely locations of oral cancers?” “In your opinion*, *which of these could cause oral cancer?” “How do you imagine oral cancers look like in the mouth?” “Can oral cancers manifest without initial complaint*, *pain*, *or symptom?” and “Is oral cancer a contagious disease?”* Participants’ answers to the 17 questions were scored as 1 for correct and 0 for incorrect responses. Each participant’s answers were summed to create a scale, “knowledge score” with 17 being the highest and 0 being the lowest. All the questions with correct answers and respondent responses are presented in a table in the results section.

### Data analysis

Bivariate association between participant characteristics and oral cancer knowledge were assessed using an independent samples t-test, one-way ANOVA and simple linear regression where appropriate. Multivariate linear regression model was used to assess the association between predictor variables and oral cancer knowledge score. All variables were included in the multivariable model regardless of their association with the outcome variable. Analyses were performed using SAS Version 9.4 (SAS Institute Inc, Cary, NC). Statistical tests were two-tailed and the significance level set at p < 0.05.

## Results

**Fig 1:**
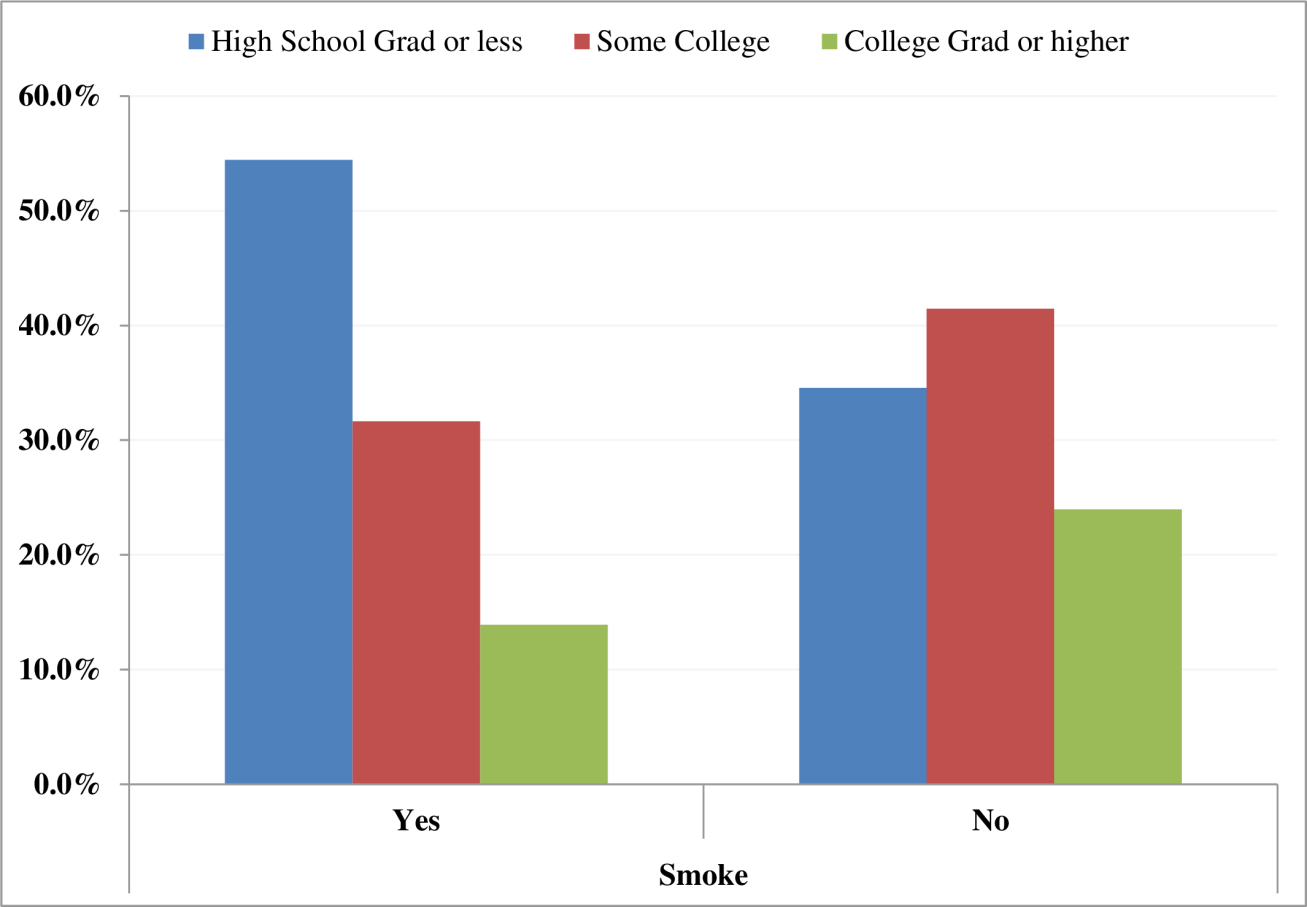
Association between tobacco use and educational status, (n = 304) using chi-square test. Chi-square test = 9.9174, *p* = 0070

A total of 304 participants (data in S1 Study Dataset) between 19 and 88 years (mean [SD], 48 [13]) completed the survey. More than 50% of participants were middle aged (45–64 years). Sixty five percent of participants identified themselves as males, 72.7% self-identified as African Americans, and 75.7% indicated they had health insurance. Smoking rate was 26.7% and 58.3% reported currently using alcohol. Mean oral cancer knowledge score was 4.6 (SD = 2.5) (Table 1). Bivariate analyses using an independent samples t-test, one-way ANOVA or simple linear regression revealed an association between age, sex, race, education and household income, and oral cancer knowledge score (Table 1). In addition, a chi-square test showed that tobacco use was significantly associated with educational status (Fig 1).

**Table 1:** Bivariate analysis of the association between oral cancer knowledge and various sociodemographic and behavioral characteristics, n = 304.

Variable	*Mean ± SD* *or n* (%)	*F*-value or t-statistic^b^	*p-*value^*^
Oral Cancer Knowledge Score	4.6 ± 2.5		
Age	48.1 ± 12.5	6.94	**.0089**
Sex		-3.37	**.0009**
Male	196 (64.5)		
Female	108 (35.5)		
Race		5.75	**.0035**
African Americans	221 (72.7)		
Caucasian	70 (23.0)		
Others^a^	13 (4.3)		
Marital status		1.06	.2916
Married/Cohabitating	149 (49.0)		
Not Married	155 (51.0)		
Education		10.73	**< .0001**
Completed high school or less	122 (40.1)		
Some college/associate degree	116 (38.2)		
College graduate	66 (21.7)		
Income		3.17	**.0245**
< $20,000	72 (23.7)		
< $40,000	67 (22.0)		
< $75,000	98 (32.3)		
≥ $75,000	67 (22.0)		
Insurance		0.66	.5119
Yes	230 (75.7)		
No	74 (24.3)		
Tobacco use		-0.79	.4313
Yes	79 (26.7)		
No	217 (73.3)		
Alcohol use		0.17	.8656
Yes	176 (58.3)		
No	126 (41.7)		

^*^P-value of .05 is considered significant and bolded.

^a^Others include Hispanic or Latin American, American Indian/Alaskan Native combined.

^b^Based on independent samples t-test, one-way ANOVA and simple linear regression test.

### Oral cancer knowledge

Table 2 contains the oral cancer knowledge questions used in the study as well as the correct answers for each question and participants’ responses. In the adjusted linear regression model, race and education remained significant predictors of oral cancer knowledge (Table 3). Compared with African Americans, Caucasians had higher oral cancer knowledge (β = 0.99; 95% CI: 0.32, 1.66). Participants with high school diploma or less had lower oral cancer knowledge compared with those with college degree or higher (β = -1.24; 95% CI: -2.44, -0.41). All other variables were not statistically significant predictors of oral cancer knowledge.

**Table 2:** Oral cancer knowledge questions and correct answers and participants response.

Questionnaire Item	Right Answer	Correct Response n (%)
*Certain types of HPV can lead to oral cancer*	True	163 (54)
*There is an HPV vaccine for both men and women that can prevent cancer*	True	112 (37)
*In your opinion oral cancers are more common in which age group?*	41 years and older	36 (12)
*In your opinion*, *in which gender are oral cancers more common?*	Men	50 (16)
*In your opinion*, *where are the most likely locations of oral cancers?*	Tongue	12 (4)
*In your opinion*, *which of these could cause oral cancer? (You may choose more than one answer)*	Alcohol, smoking, HPV	9 (3)
*How do you imagine oral cancers look like in the mouth? (You may choose more than one answer)*	Redness, ulcers, whiteness, mixture of red and white, swelling	17 (6)
*Can oral cancers manifest without initial complaint*, *pain*, *or symptom?*	Yes	151 (50)
*Does early diagnosis improve recovery from oral cancers?*	Yes	203 (67)
*Is oral cancer a contagious disease?*	No	108 (36)
*Excessive exposure to sunlight will increase a person’s chance of getting oral cancer*	No	81 (27)
*Human Papillomavirus’ infection will increase a person’s chance of getting oral cancer*	Yes	91 (30)
*Eating hot spicy foods will increase a person’s chance of getting oral cancer*	No	123 (40)
*Regular alcohol drinking will increase a person’s chance of getting oral cancer*	Yes	53 (17)
*Lack of fruits and vegetables will increase a person’s chance of getting oral cancer*	No	80 (26)
*Tobacco use in any form will increase a person’s chance of getting oral cancer*	Yes	176 (58)
*Frequently biting the cheek or lip will increase a person’s chance of getting oral cancer*	No	62 (20)

**Table 3:** Multivariate analysis of the association between oral cancer knowledge and various sociodemographic and behavioral characteristics, n = 304.

	*Oral Cancer knowledge*
	*β (95% CI)*
Age	-0.02 (-0.05, 0.01)
Sex	
Male	-0.60 (-1.18, 0.03)
Female	Reference
Race	
Caucasian	Reference
African Americans	-0.71 (-1.35, -0.07)^*^
Others^a^	1.23 (-2.26, 4.72)
Marital status	
Married/Cohabitating	Reference
Not Married	-0.14 (-0.74, 0.46)
Education	
Completed high school or less	-1.24 (-2.07, -0.41)^*^
Some college/associate degree	-0.49 (-1.24, 0.27)
College graduate	Reference
Income	
< $20,000	-0.58 (-1.51, 0.36)
< $40,000	-0.09 (-1.01, 0.83)
< $75,000	-0.29 (-1.09, 0.50)
≥ $75,000	Reference
Insurance	
Yes	Reference
No	0.28 (-0.44, 1.00)
Tobacco use	
Yes	Reference
No	0.32 (-0.34, 0.97)
Alcohol use	
Yes	Reference
No	0.14 (-0.45, 0.72)

^*^P-value of .05 is considered significant and bolded.

^a^Others include Hispanic or Latin American, American Indian/Alaskan Native combined.

## Discussion

African Americans are considered high-risk for developing tobacco-associated oral cancer [20,44]. This study aimed to quantify tobacco and alcohol rates, as well as predictors of oral cancer knowledge in a population of drag race attendees predominated by African Americans. Our results showed that smoking rate was 26.7% compared to 15.1% nationally [45]. This is consistent with previous findings that race car attendees at NASCAR smoke more compared to the general public [13,14,27]; however rate was lower than what was reported in a previous Caucasian dominated NASCAR race (31%) [40]. The high smoking rates found in our study lend support to the idea that screenings events associated with motor sports may provide an avenue for increasing awareness about main risk factors for developing oral cancer [27]. Also, significantly fewer smokers graduated from college compared with nonsmokers, supporting a previous study by White et al [40]. Our results support the notion that higher educational level is associated with diminished tobacco usage [46], and highlights the need for targeted interventions to reduce tobacco use in individuals with high school or less education.

### Oral cancer knowledge

Most of participants had lower knowledge of oral cancer, irrespective of their smoking or drinking habits, however smokers perceived their risk of developing oral cancer to be higher than nonsmokers. The association between tobacco use and health risks has been targeted in previous interventions [47], and mass media oral cancer campaigns in states such as Florida, Maryland, and North Carolina have led efforts in increasing awareness of the association between smoking and oral cancer [23,29,30]. However, more targeted awareness campaigns may be needed, tailored specifically to various populations with characteristics that make them high-risk for developing oral cancer.

### Predictors of oral cancer knowledge

Our study aimed at understanding the sociodemographic predictors of oral cancer knowledge, an important information in designing interventions to decrease cancer risk taking behaviors. After adjusting for all known covariates in our final model, there was a significant association between race, educational level, and oral cancer knowledge. African Americans traditionally have poorer oral cancer outcomes [48], and in our study, we found that they also had lower oral cancer knowledge compared with Caucasians. Since African Americans are also more likely to present with advanced stage disease than Caucasians [48], our finding of lower oral cancer knowledge in this population raises the question whether poor knowledge may be a contributing factor to late stage presentation among African Americans. If so, reducing racial disparities associated with knowledge of oral cancer could help improve early detection and increase survival rates, key elements of the *Healthy People 2020* program.

Educational status was significantly associated with oral cancer knowledge. It is concerning that participants with a high school diploma or below had lower oral cancer knowledge than those with a college degree. A previous study from New York [22] that included African Americans found that educational level predicted awareness of an oral cancer exam, but not of oral cancer knowledge. Another study that assessed whether educational level predicts oral cancer knowledge was done in an American Indian population [11]. Coupled with our other finding that higher education was associated with significantly lower smoking rate, there may be some merit in designing educational interventions specific for African Americans with a history of chronic tobacco use, and those with little education. Among African Americans, this (those with little education) may be the subgroup that would benefit the most from an oral cancer screening and education effort, given the other associated risk factors [49].

### Strengths and limitations

One of the main strengths of this study is that it is a product of a community based screening, rather than hospital based. A recent study indicates that compared to hospital-based screenings, community-based events are more effective in attracting participants with high-risk behaviors for oral cancer [27]. These high-risk behaviors have been described in the literature to include smoking and alcohol consumption, along with male predominance [49]. Based on these parameters, our study captured a high-risk group. Also, our study established that race—being African American, and educational status—having only a high school or less, are independent predictors of oral cancer knowledge among a predominantly African American population. We believe these predictors should be added to what is already known about characteristics of oral cancer high-risk groups. Attempts to identify and describe high-risk groups can help to gear screening efforts toward the populations that see greatest benefits from early cancer detection.

Some of the limitations of this study include survey limitations. Since our survey was paper based and voluntary, some of the completed surveys had some unanswered items. Participants with incomplete data were not excluded from the analysis which could lead to selection bias. However, all missing items were accounted for during data analysis; thus, we are confident that our results are not differentially skewed by the unanswered items. An additional limitation was that the sample for this study was limited to an event in one locality. Therefore, the results may not be representative of the general population of African Americans. However, we believe that since these results are from a primarily family entertainment event, it is more likely representative of the general population than if it was a hospital-based event, or even a health fair. A third limitation is the relatively small sample size. There were 3800 attendees at the drag racing event where the data was collected, but only 304 participated in the study. The fact that study and screening booth was in the vendor area, detached from the center of the racing event may explain this low number of study participants. However, this sample size is quite comparable to those from previous motor car associated oral cancer studies [13,40,50].

Future studies and educational interventions are needed in this population, and more interventions should be tailored to meet the needs of those with low educational levels, especially those with high school education or less. In addition, there is need to quantify actual oral cancer knowledge gains as a result of health education in this population; as well as to determine whether screening and educational efforts impact on tobacco and other high-risk behaviors. Future studies could also explore how educational leaflets [24,51] and other education strategies [52] tailored specifically to this population could lead to increase oral cancer knowledge. Finally, since African Americans are also more likely to present with advanced stage disease than Caucasians [48], future studies should investigate how poor oral cancer knowledge correlate with stage of presentation among oral cancer patients, stratified by race and other key factors, including gender.

## Conclusions

Oral cancer knowledge is generally poor in the community and worse among the African Americans in our study population. With the changing landscape in oral cancer epidemiology, it is important that factors that predict oral cancer knowledge and risk perception be incorporated when designing interventions to prevent and detect oral cancer among African Americans and other high risk groups.

## Supporting information

S1 Study Dataset Analytical dataset used in the study. (SAS7BDAT)
